# Modelling the potential impact of mask use in schools and society on COVID-19 control in the UK

**DOI:** 10.1038/s41598-021-88075-0

**Published:** 2021-04-22

**Authors:** J. Panovska-Griffiths, C. C. Kerr, W. Waites, R. M. Stuart, D. Mistry, D. Foster, D. J. Klein, R. M. Viner, C. Bonell

**Affiliations:** 1 Department of Applied Health Research, University College London, London, UK; 2 Institute for Global Health, University College London, London, UK; 3 The Wolfson Centre for Mathematical Biology and The Queen’s College, Oxford University, Oxford, UK; 4 Institute for Disease Modeling, Global Health Division, Bill & Melinda Gates Foundation, Seattle, WA USA; 5 School of Physics, University of Sydney, Sydney, NSW Australia; 6 School of Informatics, University of Edinburgh, Edinburgh, UK; 7 The Centre for the Mathematical Modelling of Infectious Diseases, the London School of Hygiene & Tropical Medicine, London, UK; 8 Department of Mathematical Sciences, University of Copenhagen, Copenhagen, Denmark; 9 Disease Elimination Program, Burnet Institute, Melbourne, VIC Australia; 10 University of Sheffield, Sheffield, UK; 11 UCL Great Ormond St. Institute of Child Health, London, UK; 12 Faculty of Public Health and Policy, London School of Hygiene and Tropical Medicine, London, UK

**Keywords:** Epidemiology, Computational science, Computational models, Health policy

## Abstract

As the UK reopened after the first wave of the COVID-19 epidemic, crucial questions emerged around the role for ongoing interventions, including test-trace-isolate (TTI) strategies and mandatory masks. Here we assess the importance of masks in secondary schools by evaluating their impact over September 1–October 23, 2020. We show that, assuming TTI levels from August 2020 and no fundamental changes in the virus’s transmissibility, adoption of masks in secondary schools would have reduced the predicted size of a second wave, but preventing it would have required 68% or 46% of those with symptoms to seek testing (assuming masks’ effective coverage 15% or 30% respectively). With masks in community settings but not secondary schools, the required testing rates increase to 76% and 57%.

## Introduction

Evidence to date suggests that SARS-CoV-2, the coronavirus that causes COVID-19, is mainly transmitted when someone who has COVID-19 coughs, sneezes or exhales and releases droplets of infected fluid^1^. Some of the droplets can be breathed in by people within a close proximity and some will fall on nearby surfaces and objects. If people touch the contaminated objects and then touch their eyes, nose or mouth, COVID-19 may also be transmitted^2,3^. Reducing the frequency of physical contact, maintaining good hygiene and good ventilation, and pursuing effective test-trace-isolate (TTI) strategies are important non-pharmaceutical interventions that can reduce COVID-19 transmission and have been used worldwide since the onset of the pandemic. They will continue to form an important adjunct to national prevention strategies together with continual vaccination roll-out.

In the early stages of the COVID-19 pandemic, there was uncertainty over the effectiveness of face coverings in reducing the spread of COVID-19 and protecting the public^4,5^. While public use of face coverings was adopted early in many Asian countries that have had experience with epidemics such as SARS in 2003^6^, countries such as the USA and the UK were slower in mandating face coverings, despite relatively high levels of public support^7^. For the purposes of this study, we will refer to face coverings or masks interchangeably to mean face protection that covers the mouth and nose.

There is now considerable evidence supporting the effectiveness of masks for protecting against transmission between individuals. Laboratory experiments have found that almost all types of masks can greatly reduce droplet emission and viral shedding by infectious wearers^8,9^, suggesting their effectiveness for source control. Two observational studies^10,11^ and recent systematic reviews focusing on SARS-1, MERS and influenza^12–15^ indicate that masks also substantially reduce infection risk to the non-infected wearer, even when their infectious contact is unmasked. Specifically, Chu et al.^12^ suggest that the use of a surgical or cotton mask could result in a reduction in infection risk of around 44% (95% CI 11–60%) in a community setting, with stronger associations in a healthcare setting (70% [59–78%]) and using an N95 respirator (96% [70–99.6%]). A randomised controlled trial (RCT) was conducted in Denmark over April-May 2020 to explore the impact of face coverings against COVID-19 infection, but results were inconclusive owing to the limitations of the study stemming from missing data, reliance on patient-reported data, and the confounding effect of other interventions^16^. Observational studies are prone to confounding, and RCTs focused on influenza are inconclusive^13^. Epidemiological studies have also shown a negative association between mask prevalence and COVID-19 incidence at a city, state and national level^17–20^.

Since mid-2020, the use of face coverings became mandatory in community settings across the four UK nations. In England, masks became mandatory on public transport on June 15, 2020, and in shops from July 24, 2020, with further expansion of places where masks are mandatory announced on July 31, 2020^21^ and new rules being enforceable by law from August 08, 2020. In Scotland, masks became mandatory on public transport from June 22, 2020 and in shops from July 10, 2020^22^. Masks became mandatory on public transport in Wales and Northern Ireland from July 10, 2020^23^.

However, the magnitude of the effect of compulsory masking on COVID-19 incidence is still highly uncertain since there is no straightforward way to infer a change in transmission probability from a change in viral emission. Mathematical modelling, having already played an important role in informing policy around the COVID-19 pandemic^24–31^, can help to assess the likely impact of compulsory masking. Models have been used to evaluate the impact of the lockdown^29,30^, to explore the efficacy of different test-trace-isolate (TTI) strategies^26–28,31^ and to consider the impact of masks on the COVID-19 epidemic^32–39^. While the overall message from these models is that, as lockdown measures are relaxed, masks are likely to be effective if they are worn by a large percentage of the population, they often optimistically assumed that a large proportion of transmission events could be prevented with face masks by overestimating their efficacy and guided by evidence of the impact of masks on influenza transmission reduction^15^; hence their results are likely to overestimate impact. Furthermore, with the exception of a few studies^36–39^, many studies to date have used population-based models that do not differentiate between household, school, workplace and community contacts. The UK policies on masks that were in effect in August 2020 only affected community contacts, so untangling different layers of contacts is crucial in evaluating the impact of masks. In summary, existing modelling studies are likely to exaggerate the impact of masks.

**Figure 1:**
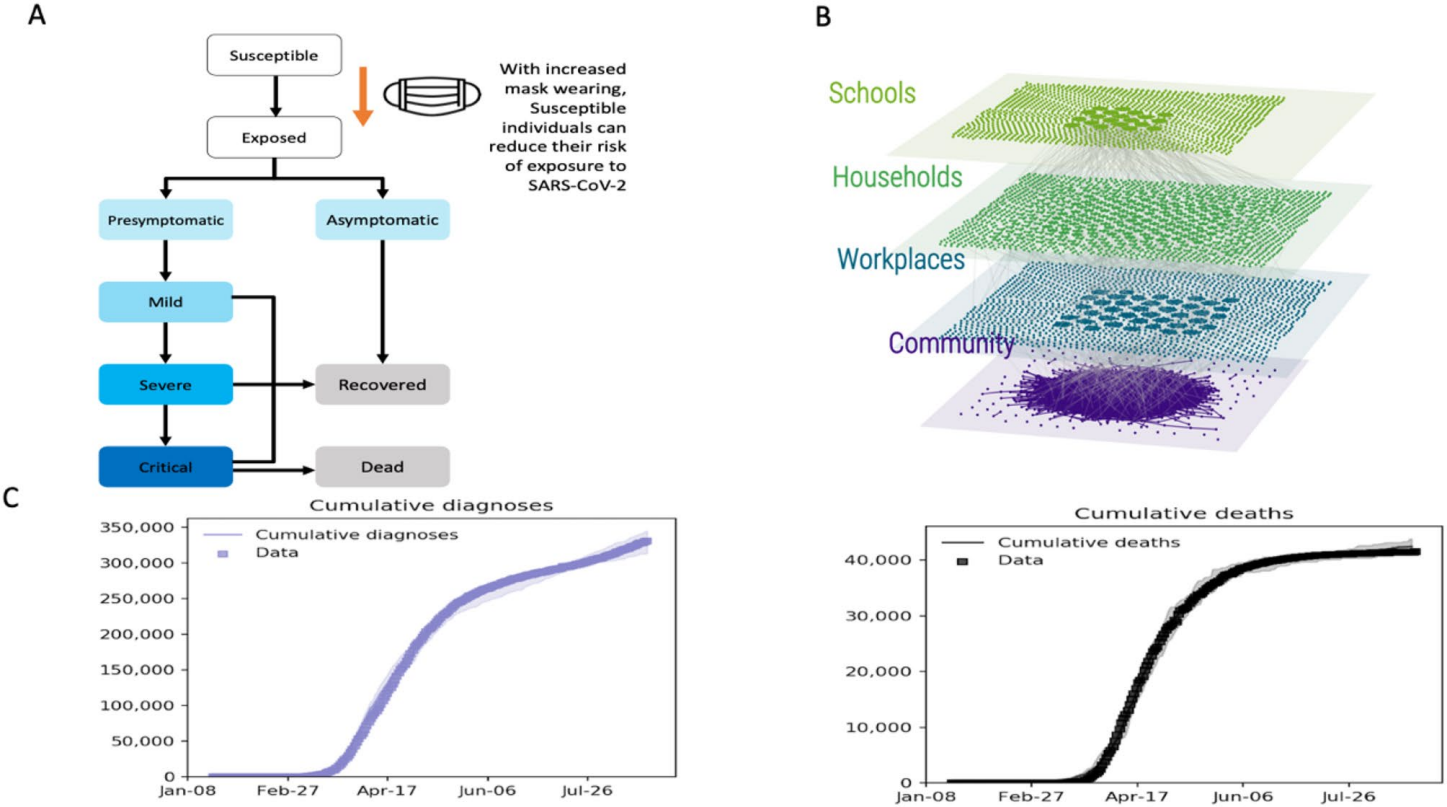
(**A**) Schematic of the model component showing the modelled effect from using face coverings. (**B**) Illustration of the different levels modelled in Covasim. (**C**) Results of the model calibration showing the matching of the model projected cumulative COVID-19 cases and cumulative deaths associated with COVID-19 with the data from https://coronavirus.data.gov.uk. Data is shown in thick blue/black lines, medians across twelve simulations are indicated by thin blue/blue lines and 10% and 90% quantiles by blue/grey shading.

Our previous work^28^, which used the detailed individual-based model called Covasim^36^, highlighted that adequate TTI would be needed to prevent a secondary epidemic wave following the reopening of broader society, including schools, in the UK after the first epidemic wave. Here, we extend this work by calibrating Covasim to August 28, 2020 (Fig. 1A,B), taking in consideration the slower-than-anticipated reopening of society in the UK during July and August, and exploring whether extending mandatory mask use to secondary school students alongside community settings could have contributed to reducing the risk of COVID-19 wave resurgence from September 2020. The findings from the CoMIX studies suggested that the contact rate in England was increasing much slower than anticipated over July and August of 2020, with an average of 4 contacts per person compared to around 11 contacts per day in pre-COVID-19 era^30^. We anticipate that this will increase with reopening of schools but less than the anticipated 90% of the pre-COVID-19 contact rate previously modelled in^28^.

WHO recommends mask use for children over 12 years under the same conditions as adults, with decisions on mandatory use for those between 6 and 11 based on a set of conditions^40^. This recommendation is on the basis that younger children may have lower susceptibility and potentially lower transmissibility than adults^41^. While the evidence is not conclusive, we anticipate the practicalities with younger children correctly obeying masks rules are more challenging than for older children. In addition, face masks are more likely to hamper the education of primary than secondary school children given the focus on learning basic speaking and social skills. Children over 12 years old in the context of the UK school systems attend secondary schools and in mid-2020, following WHO guidance^42^, the government permitted schools to mandate mask wearing in communal areas (where high levels of social mixing and lower levels of social distancing may occur)^43^. Overall data on mask use in children is sparse, with a WHO document^40^ suggesting that mask fit and compliance is likely to be poorer in children than in adults and hence mask efficacy levels based upon adult data may need to be adjusted. To account for this uncertainty in the compliance with mask-wearing and efficacy, we simulate two levels of masks’ effective coverage in schools and community settings, estimated as the product of the masks' efficacy (per-contact risk reduction) and coverage (the proportion of contacts in which they are worn); see “Methods” section). We estimate the different combinations of testing and tracing levels necessary to avoid COVID-19 resurgence after September 2020 (when schools reopened in the UK), accounting for community mask-wearing and scenarios with and without mandatory masks in secondary schools, including two levels of masks’ effective coverage. The strategies we have explored were discussed and results shared with members of scientific advisory bodies in the UK, including the Scientific Pandemic Influenza Group on Modelling (SPI-M).

## Results

As part of calibrating the model, we ran 3000 simulations, filtered these to retain the 1% (30 simulations) which gave the best fit to the data, and used these to make our future estimates. During the calibration we estimated the daily probabilities of testing people with symptoms between January 21, 2020 and August 28, 2020. These were 2.77% for both July and August, corresponding to around 24% of people with symptomatic infection tested during their illness (assuming an average symptomatic period of around 10 days). We note that this is higher than the 18% testing level when we calibrated until June 17, 2020 in^28^. Furthermore, in calibrating to the UK epidemic, we estimated that 1500 people were infected in the UK on January 21, 2020, that the per-contact transmission probability was 0.75% under the assumption that 70% of infections were symptomatic, and that 0.076% in May–August 2020 and 0.28% after August 2020 of those with asymptomatic COVID-19 infections were tested at some point during their illness (see “Methods” section for details). Figure 1C shows the results of the model calibration.

**Figure 2:**
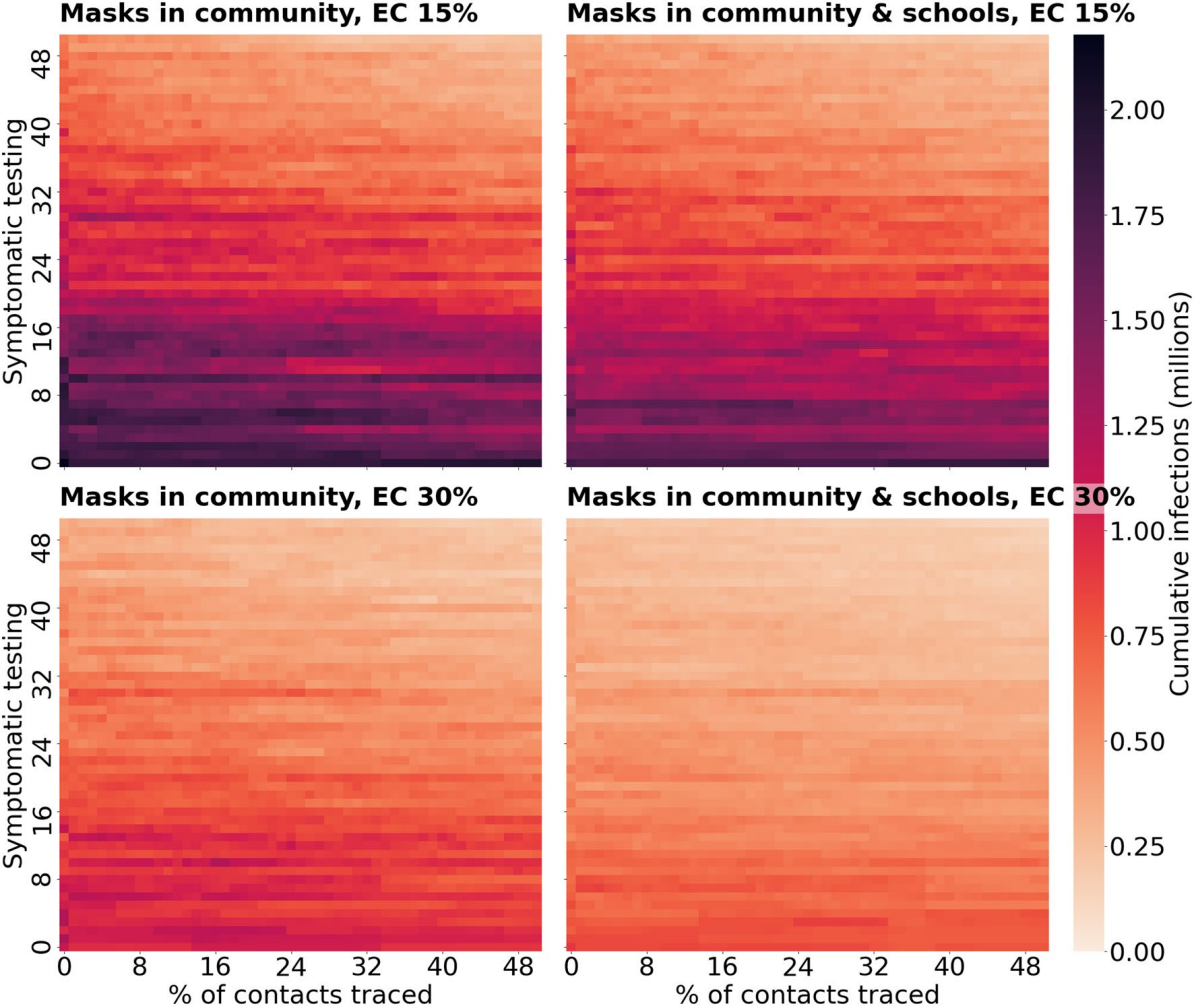
Heatmaps of cumulative infections for different trace (x-axis) and test (y-axis) levels across the scenario of mask wearing in parts of community with and without schools masks’ wear. Higher cumulative infections are shown in darker shades of red, while lower values are lighter colours. The region of a light orange colour where cumulative infections remain below 500,000, represents a region within where the second wave of COVID-19 after September 2020 is avoided with combinations of adequate test-trace and mask usage.

**Figure 3:**
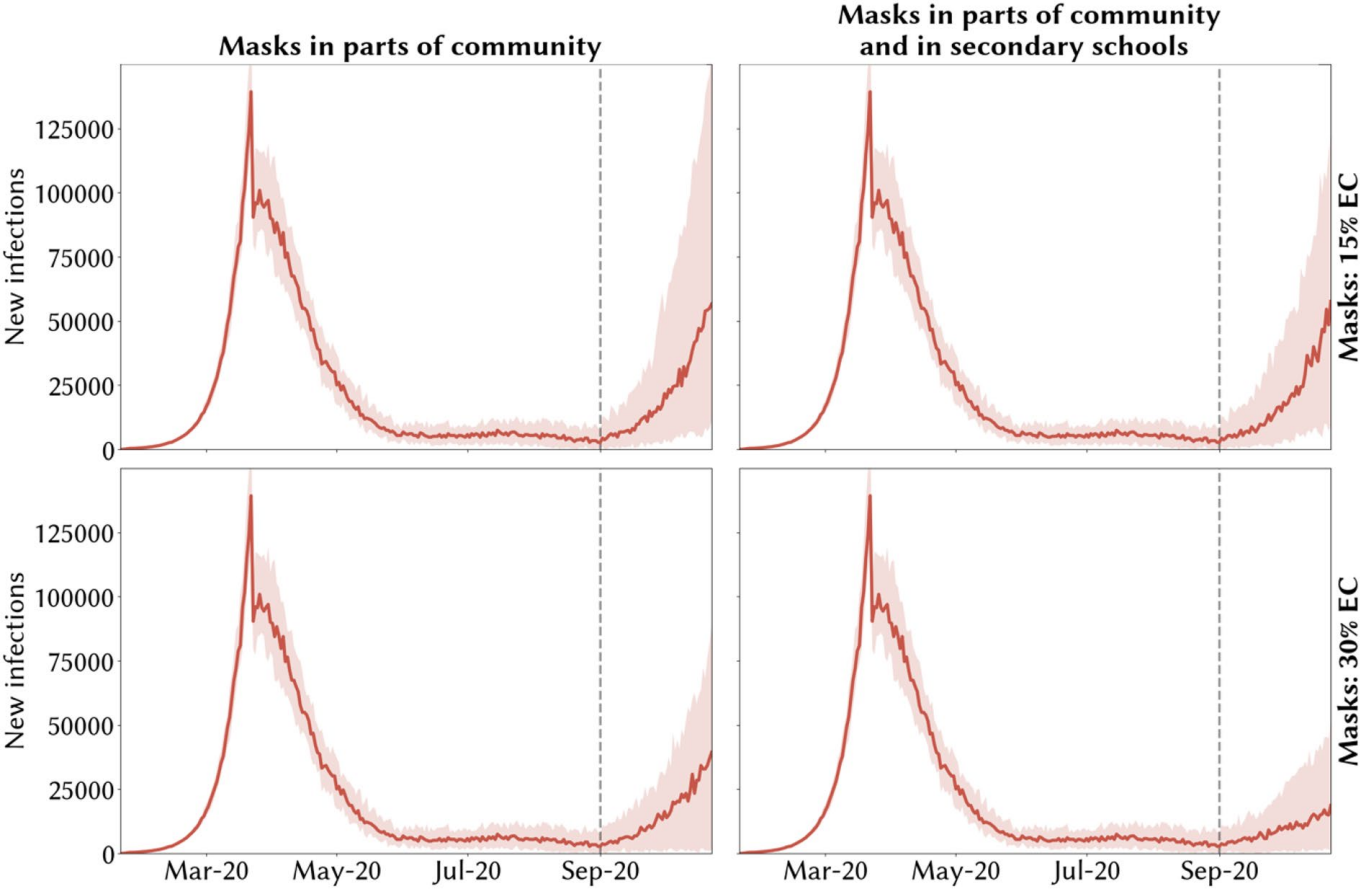
Model scenarios of potential second COVID-19 epidemic wave in the UK during 2020 under different policies of mask wearing and effective coverage, assuming a testing level of 24% and school/workplace tracing levels of 50%. Medians across 30 simulations are indicated by solid red lines and 10% and 90% quantiles by red shading. The resurgence of COVID-19 after September 2020 is controlled and second wave avoided with combinations of adequate test-trace and mask usage if the median number of daily infections remains below 20,000 over the simulation period.

**Figure 4:**
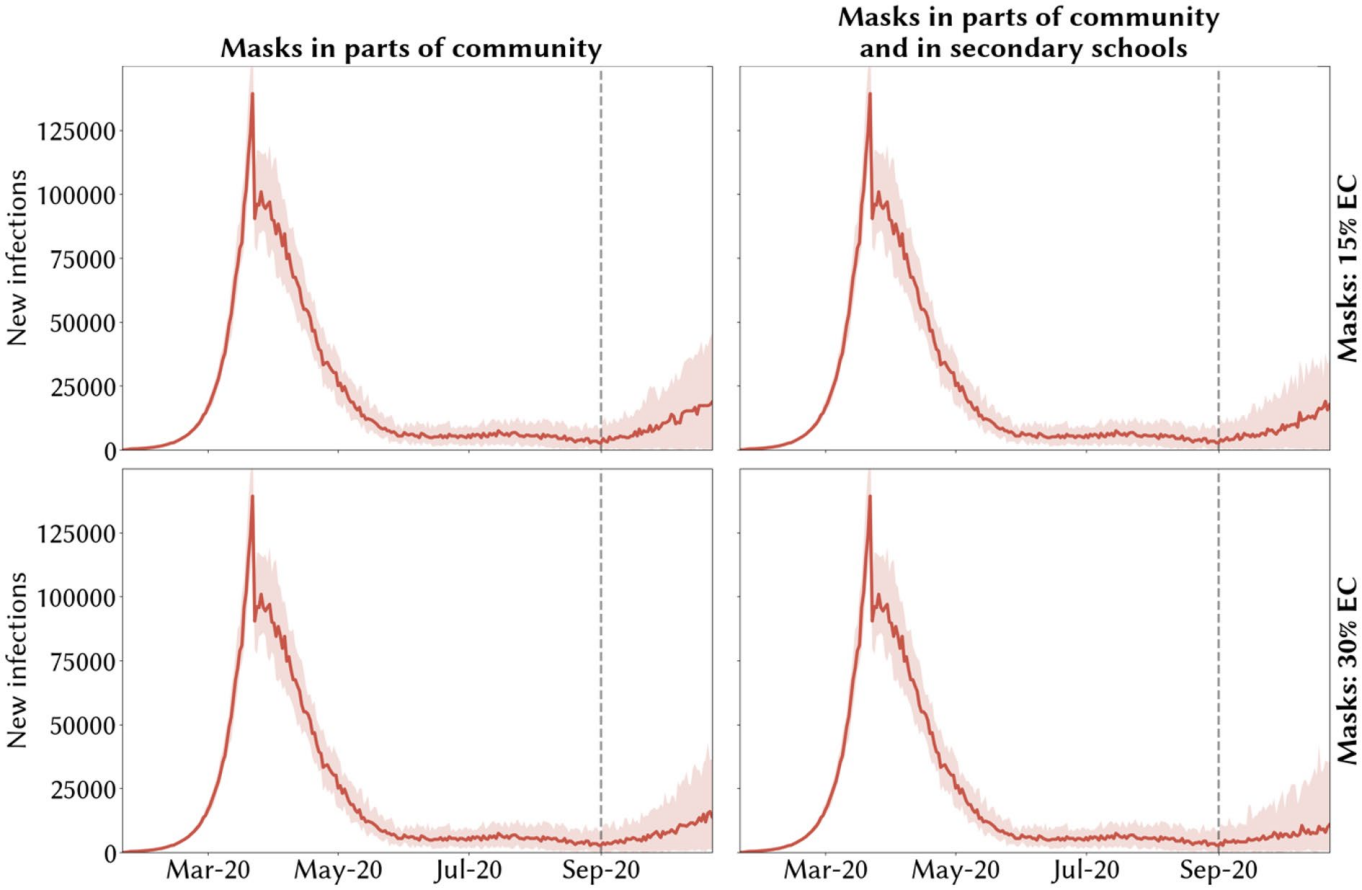
Model scenarios of potential second COVID-19 epidemic wave in the UK during 2020 under different policies of mask wearing and effective coverage for testing and tracing levels where future the resurgence of COVID-19 is prevented. Medians across 30 simulations are indicated by solid red lines and 10% and 90% quantiles by red shading. The resurgence of COVID-19 after September 2020 is controlled and second wave avoided with combinations of adequate test-trace and mask usage if the median number of daily infections remains below 20,000 over the simulation period.

The projections from the calibrated model across all scenarios are shown in Figs. 2, 3 and 4. In Fig. 2 we show heatmaps of cumulative infections for different trace (x-axis) and test (y-axis) levels. Increases in cumulative infections result from resurgences of COVID-19; comparable increases can be seen in the peak of new infections (Fig. S1). In Fig. 2, higher cumulative infections are shown in darker shades of red, while lower values are lighter colours. Across these figures (and respectively Fig. S1 of the supplementary material), a very light orange colour represents a region within the heatmaps where the resurgence of COVID-19 after September 01, 2020 has median daily infections less than 20,000 (Fig. S1) and median cumulative infections below 50,000 by the end of the simulation period (Fig. 2). We refer to this parameter regime as being a parameter regime where the second COVID-19 wave is avoided and the resurgence controlled with combinations of adequate test-trace and mask use.

In Figs. 3 and 4, across the four scenarios, we show illustrative temporal profiles for the new infections for two different combinations of test-trace levels: the current 24% testing and 50% tracing level. In Fig. 3 a second wave is predicted after September 01, 2020 with median daily infections more than 20,000 over the simulation period, while in Fig. 4 with enhanced test-trace combination and effective masking the median daily infections remain less than 20,000 over the simulation period and resurgence of COVID-19 is avoided.

### Masks worn in some community settings but not in secondary schools

We estimate that reopening of broad areas of society in the UK together with schools from September 1, 2020, with masks mandatory in parts of community but not in schools, would result in an increase in COVID-19 cases if test-trace levels are insufficient under both assumptions about masks’ effective coverage (dark red region in Fig. 2A,C). Using testing levels from August 2020 (24% of symptomatic people tested at some point of their infection) and with 50% of contacts traceable, we predict an increase in the number of new COVID-19 infections from September 2020 over 20,000 per day under both assumptions of masks’ effective coverage (Fig. 3A,C). The strength of the secondary COVID-19 wave is predicted to vary depending on the effective coverage of masks (Fig. 3A,C).

With adequate combinations of test-trace levels, a second epidemic wave may be avoided, with cumulative infections remaining below 500,000 cases and daily infections below 20,000 cases (Fig. 2A,C, Figure S1A,C within light orange-coloured region). For example, to achieve this, assuming 50% of contacts could be traced, similar to the levels in July and August 2020, with low mask effective coverage (15%) in relevant community settings, 76% of those with symptomatic infection would need to be diagnosed and isolated (Fig. 4A). If masks’ effective coverage in community settings were higher (30%), the necessary testing level would be 57% (Fig. 4C).

### Masks worn in secondary schools alongside some community settings

With reopening of broad areas of society in the UK together with schools from September 1, 2020, with masks mandatory in parts of community and in secondary schools, our model predicts a secondary COVID-19 wave, represented by daily infections increasing above 20,000 in Fig. S1 and cumulative infections increasing above 500,000 infections over the simulation period in Fig. 2, would occur in the absence of an adequate test-trace program. This is the case under both assumptions of masks’ effective coverage (dark red region in Fig. 2B,D). For the scenario of low (15%) effective coverage of masks, under testing (24%) and tracing levels (47%) from August 2020, the model predicts that a resurgence in COVID-19 cases would be likely to start from September 2020 (Fig. 3B). However, with higher (30%) effective coverage of masks, and if they are mandatory in secondary schools, the strength of the second epidemic wave is predicted to be much less. This is evident from comparing the size of the light orange region in Fig. 2B,D and also when comparing Fig. 3B,D for a single combination of testing and tracing levels.

With a scaled-up version of the TTI strategy in place in August 2020, we estimate that the resurgence in COVID-19 with daily cases increasing above 20,000 infections could be avoided (Fig. 4B,D). To achieve this, assuming August 2020’s tracing levels (47% of contacts traced and isolated) were to continue, we estimate that it would be necessary to test 68% or 46% of those with symptomatic infection during their infectiousness period, assuming masks’ effective coverage of 15% or 30% respectively.

### Masks in secondary schools vs masks not in secondary schools

Our results suggest that there is a greater benefit of mandatory masks in secondary schools if the effective coverage of masks is high (30%) (Fig. 3A vs B, C vs D). Under August 2020’s testing and tracing levels (24% testing, 47% tracing) and masks’ effective coverage of 30%, the predicted second COVID-19 wave would be less than half of the original wave if masks were mandatory in secondary schools as well as in community settings (comparing Fig. 3C,D). If the effective coverage of masks is less (15%), the effect of the mask wearing in schools on the predicted wave is much less (comparing Fig. 3A,B). The minimum testing levels necessary to avoid a second wave, under scaled up TTI, is 8–11% less when masks are mandatory in schools than if they are not, depending on the effective coverage of masks (76% and 57% in Fig. 4A,C compared to 68% and 46% in Fig. 4B,D respectively).

## Discussion

Our results suggest that, with broader society including schools reopened from September 2020, and with levels of coverage of TTI as in August 2020, mandating the use of masks in secondary schools would result in fewer infections, but would not be sufficient to prevent a COVID-19 resurgence in late 2020, even without assuming any changes in the fundamental characteristics of the virus. Only with increased TTI coverage could a such resurgence of COVID-19, with daily cases increasing above 20,000 and cumulative cases over 500,000 over the simulation period, be avoided. The necessary TTI coverage requires testing sufficient number of symptomatic people and then sufficient identification, tracing and isolation of their positive contacts. Across different assumptions of masks’ effective coverage, different levels of testing and tracing would be necessary to avoid a second epidemic wave. For example, if masks were mandatory in secondary schools and tracing continued at its August 2020 level of 50%, 68% or 46% of those with symptomatic infection would need to be tested respectively under scenarios of 15% and 30% mask effective coverage. If masks were not mandatory at secondary schools, the respective numbers would be 76% and 57% for 15% and 30% effective coverage of masks in community settings.

Overall, our findings suggest that making masks mandatory in secondary schools would be of benefit but would need to be combined with scaling up of TTI coverage to prevent resurgence of COVID-19. We highlight that adoption of masks in schools, in addition to using in community settings, can help reduce epidemic resurgence, but, to do this effectively, the effective coverage of masks has to be assumed to be sufficiently high (30% in our case). If it were lower, the reduction in the estimated COVID-19 resurgence would be smaller. Uncertainties concerning the effectiveness of the masks remain, and these results add to the ongoing body of evidence on the impact of using face masks against epidemic spread.

Unlike previous work that has considered specific values of testing level and projecting outcomes, our modelling provides all combinations of thresholds for testing and tracing coverage that could prevent a COVID-19 resurgence whether or not masks were mandatory in secondary schools. Furthermore, previous studies^32–39^ have explored the broader impact of masks by simulating lowered transmission across all layers in society. By contrast, the Covasim model has the granularity to consider specific layers, hence allowing our study to specifically explore the impact of mandatory masks in secondary schools, in combination with different levels of TTI coverage.

The analyses presented here have several limitations. First, while we have made an effort to model the UK epidemic, some of the parameters we have used are from other settings. Secondly, as with any modelling study, we have made a series of assumptions within the modelling framework, for example concerning the proportion of infections which are symptomatic, and the susceptibility and infectiousness of children compared to adults. Large uncertainty regarding the proportion of asymptomatic infection remains with recent evidence^44^ suggesting that asymptomatic incidence has a wide range of 2–57%. In our model, we have assumed that symptomatic infections account for 70% of all infections and that development of symptoms is age-dependent, analogous to other studies^26–29^.

The model considers mask wearing to be uniform in the sense that, for a given level of coverage in a subpopulation (e.g. a school), the average transmission probability is reduced by the same amount. In reality, there are four possible cases for an interaction between a susceptible and an infectious individual: both are wearing a mask, neither are wearing a mask, or one or the other is wearing a mask. The transmission probability will be different for each of these four cases. Because the way in which transmission probability can be expected to vary is not well-understood, we use ‘effective coverage’ as a catch-all for the four different cases. More research is required to understand the extent to which these fine-grained details matter and work on this is ongoing.

Our simulations over the period from September 2020 onwards reflect a great deal of uncertainty, as can be seen from the shaded bands in Figs. 3 and 4. Each of our model simulations represents one possible realisation of the flow of COVID-19 transmission among the population, but since this depends on the exact characteristics of who gets infected and when, the dynamics of transmission over long periods are subject to substantial uncertainties. While we have captured some of this uncertainty in our epidemic projections, we have not captured the impact of this on our estimates of the testing levels required to avoid a secondary epidemic wave, nor on our estimates of the population-level efficacy of masks. Also, we have provided point estimates of the testing levels that would be necessary to prevent resurgence, but these should be interpreted with a degree of caution since they are based on the median simulation from a stochastic process with a degree of variation and future changes such as behavioural changes, possible emergence of virus variants and effects from future vaccine roll-out would affect these estimates.

Finally, we note that we do not account for interactions between regions that differ in policy and infrastructure; for example the differences between Scotland’s ‘Test and Protect’ strategy and the NHS England ‘Test and Trace’ strategy. Rules on distancing and mask wearing have also been varied in different regions where prevalence is higher. We do not, however, attempt to model the effect of individuals moving between regions mediating the interaction of these different epidemics unfolding in different ways under different conditions. These aspects are being considered as extension of this work.

Under the policy on masks in UK in effect in August 2020, masks were recommended in workplaces, but there are some variations in policy recommendations across the four UK nations on these e.g. workplaces that are open to the public requiring the workers to wear masks in earlier in Scotland that in England^22^. Since our intention was to model the UK epidemic as a whole, we do not differentiate between these differences. Instead, the assumption of 50% transmission remaining in workplaces, is based on including both proportion of people remaining working from home from September 2020^45^, but also NPIs within workplaces e.g. masks wear and hygiene and social distancing policies within workplaces.

We have modelled scenarios in which masks would be worn by secondary school children only since this was the policy recommendation being followed in the UK as of August 2020, informed by evidence that younger children have lower susceptibility and potentially lower transmissibility than adults^41^, and observational evidence that many of the school-based outbreaks observed globally have been concentrated in older students^46^. In the process of developing our models and preparing this manuscript, we have explored additional scenarios with masks mandatory in all schools, only primary schools etc., but the results of these other models are not included here as these were not under consideration for UK policy on masks at the time of writing. Within the modelling framework, we can evaluate a large number of scenarios considering different permutations of mask usage by different cohorts and across various settings. But the purpose of this study was to evaluate a feasible and policy-relevant subset of these permutations.

The UK’s plans for reopening schools in September 2020 were accompanied by numerous countermeasures relating to physical distancing, hygiene, ventilation and ‘bubbles’ of year groups in secondary schools and classes in primary schools as well as masks. With these countermeasures in place, it is expected that school-based transmission risk to students, teachers and staff may be somewhat mitigated, but the adherence to these policies by school children at all times is uncertain. Hence on balance, for this analysis, we have assumed that such measures could reduce transmission by 10%. We note that this is a modelling assumption and it may be an underestimation.

Furthermore, while our findings suggest that adequate TTI is required alongside masks wearing in secondary schools and in community settings may be able to reduce the strength and prevent COVID-19 resurgence, the increased numbers of contacts due to school mixing, and reduced adherence to school-based rules on social distancing, both within and outside of schools, can easily overwhelm the TTI programs. The questions remain whether the UK has sufficient testing capacity to (a) scale-up to levels suggested by our study and (b) trace increasing numbers of contacts per index case in schools, workplaces and community settings. We acknowledge that achieving this will be challenging, and especially so if the UK is faced with resurgences with possible test delays, test capacity being reached and the contact tracing system being overwhelmed. In addition, effective isolation is also important and the currently reported levels of isolation need to be improved^47,48^. All of these factors could further reduce the impact of the TTI programme.

Overall, our findings suggest that mandating masks in secondary schools in addition to other parts of society could reduce the strength of COVID-19 resurgence in the UK. Wearing masks forms a barrier for the viral particles to pass from the wearer to people surrounding them and vice versa^42^, and hence wearing them at schools could reduce COVID-19 incidence in students, staff and teachers.

In summary, our modelling suggests that while adoption of masks in secondary schools in addition to community settings may contribute to reducing the size of a second wave, it would not be sufficient to prevent a secondary COVID-19 wave in the UK, even without considering the impact of new, potentially more transmissible variants of the virus. Instead, a masks policy would need to be combined with adequate TTI strategy that can test a large proportion of symptomatic people during their infectious period, effectively trace their contacts and isolate them.

## Methods

### Transmission model

Analogous to our recently published work^28^, we modelled the spread of COVID-19 using Covasim, a stochastic individual-based model of SARS-CoV-2 transmission across a population. Development and implementation details can be found at http://docs.covasim.org with the methodology outlined in^36^. The model was previously applied to explore different scenarios of schools reopening in the UK^28^, explore the epidemic spread in Australia^37^ and explore different non-pharmaceutical interventions for epidemic control in Seattle, USA^36^. The code used to run all simulations contained in this paper is available from https://github.com/Jasminapg/Covid-19-Analysis.

For the purposes of the analyses presented in this paper, we used Covasim’s default parameters together with demographic data on population age structures and household sizes for the UK, with four population contact network layers for schools, workplaces, households and community settings. The per-contact transmission probability (the risk of SARS-CoV-2 transmission during a contact between an infectious individual and a susceptible individual) is assumed to depend on the contact network. Covasim accounts for testing strategies via parameters that determine the probabilities with which people with different symptoms receive a test each day.

### Data sources and calibration

We used Covasim to generate a population of 100,000 agents who interact over the four contact networks layers described above. To reflect the size of the UK population, we used dynamic scaling, as described in details in^36^, of these 100,000 agents up to the population of around 68 million. This methodology allows cumulative number of cases generated from infection emerging from these agents to be modelled over time. Furthermore, within individual-based-models such as Covasim, dynamic scaling allows for arbitrarily large populations to be modelled whilst maintaining a constant level of precision and manageable computation time throughout.

To fit the model to the UK epidemic, we performed an automated search for the optimal values of the number of infected people on 21 January 2020, the per-contact transmission probability, and the daily testing probabilities for symptomatic individuals (${p}_{s}$) during May, June, July and August. The optimal values were the ones that minimised the sum of squared differences between the model’s estimates of confirmed cases and deaths, and data on these same two indicators between January 21, 2020 and August 28, 2020 collated from the UK government’s COVID-19 dashboard (https://coronavirus.data.gov.uk). These particular parameters were selected as the most important to estimate because of the considerable uncertainties around them. We accounted for effect of the lockdown by reducing the per-contact transmission probabilities from 23 March 2020, up to 2% of their pre-lockdown values within schools, and to 20% of their pre-lockdown values within workplace and community settings, and increased these in a phased way since the phased relaxing of the lockdown measures from 1st June. Exact scaling factors are shown in Table 1.

**Table 1:** Scale factors applied to daily “pre-lockdown” SARS-CoV-2 transmission probabilities in households, schools, workplaces from September 2020, and the community under the scenarios of face coverings policy and compliance levels.

Scenarios	Masks’ effective coverage scenarios				
	Assumptions	Household contacts	School contacts	Work contacts	Community contacts
Masks in community not in secondary schools with low compliance	50% coverage of face coverings in community 30% efficacy of face coverings Masks not in secondary schools Masks not in workplaces	100%	90% from 1st September and during term-time 2% during school holidays	50% from 1st September and during term-time 40% during school holidays	60% from 1st September and during term-time 51% during school holidays
Masks in community not in secondary schools with high compliance	50% coverage of face coverings in community 60% efficacy of face coverings Masks not in secondary schools Masks not in workplaces	100%	90% from 1st September and during term-time 2% during school holidays	50% from 1st September and during term-time 40% during school holidays	49% from 1st September and during term-time 42% during school holidays
Masks in community and in secondary schools with low compliance	50% coverage of face coverings in community 30% efficacy of face coverings 50% coverage of face coverings in schools (only secondary schools) Masks not in workplaces	100%	77% from 1st September and during term-time 2% during school holidays	50% from 1st September and during term-time 40% during school holidays	60% from 1st September and during term-time 51% during school holidays
Masks in community and in secondary schools with high compliance	50% coverage of face coverings in community 60% efficacy of face coverings 50% coverage of face coverings in schools (only secondary schools) Masks not in workplaces	100%	63% from 1st September and during term-time 2% during school holidays	50% from 1st September and during term-time 40% during school holidays	49% from 1st September and during term-time 42% during school holidays

Scale factors applied prior to September 2020 are the same across all scenarios and are summarised in Table S4.

We also used publicly available weekly data from NHS Test and Trace to generate a level of contact tracing of contacts of those testing positive since the start of the programme on May 28, 2020 and collated in Table 2. We multiplied the percentage of people testing positive that were interviewed, the percentage of those reporting contacts and the percentage of contacts that were traced to generate an overall percentage for contacts of those tested positive that were traced. This assumed that all those testing positive would have the same number of contacts regardless of whether or not these were reported. We determined that this contact tracing level was 42% for June, 47% for July and 44% August, with an average of 50% tracing since 28th May 2020. The model differentiates tracing levels depending on layer considered, and for this study we assumed that 100% of household contacts could be traced within the same day of a positive diagnosis and 5% of community contacts could successfully be traced within 2 days. We then varied the level of contact tracing in schools and workplaces to change the average level of monthly tracing in our analysis. Under the current scenario we assumed that 50% of contacts within schools and workplaces were traced within 1 day and this produced an average monthly tracing level comparable with reported monthly values after June 2020 from^24^. We also assumed that asymptomatic testing is available across all society layers and modelled this in line with reported numbers in the UK (0.076% May–August 2020, 0.28% after August 2020 from https://ourworldindata.org/coronavirus-testing). Finally, based on discussions with scientific and policy decision makers in the UK, we assumed that 30% of people adhere to the recommended isolation period of 10 days.

**Table 2:** Levels of contact tracing during June 2020, July 2020 and August 2020 collated from NHS Test and Trace available at^49^.  from weekly report from NHS Test and Trace available from^49^.

Weeks	% of people tested positive that are reached within 1–2 days	% of people tested positive that provided 1 or more close contacts	% of close contacts reached within 1–2 days	
28th May–24th June	73.9	67.6	86.4	^49^
25th June–1st July	77.4	75.8	70.8	^49^
2nd July–8th July	78.7	78.2	71.1	^49^
9th July–15th July	79.7	79.9	77.9	^49^
16th July–22nd July	81.4	81.3	75.1	^49^
23rd July–29thJuly	79.4	79.7	72.4	^49^
30th July–5th August	79.7	78.6	71.4	^49^
6th August–12th August	78.8	77.6	71.3	^49^
13th August–19th August	72.6	75.9	75.5	^49^
20th August–26th August	81.4	80.2	69.4	^49^

### Modelled effective coverage of different contact network layers

In this study we used effective coverage as a measure for effectiveness across all types of face coverings, and we translated this into the modelling framework by reducing the transmission probability of different population layers.

We assumed that masks were mandatory in parts of community, such as public transport from June 15, 2020, and then extended to more places, such as shops, from July 24, 2020. Depending on the scenario, we then additionally assumed that masks would either be mandatory throughout all secondary schools from September 1, 2020, or that they would remain optional depending on schools’ specific policies (see below). For each scenario, we derived effective coverage of face coverings as a product of efficacy and coverage (proportion of contacts in which at least one mask is used) across layers. We modelled these policies by scaling down the probability of transmission by this effective coverage level in the relevant layers depending on the two policies of face coverings with details in Table 1. We used two values of effective coverage to describe a lower and higher effective coverage of face coverings across settings; below we describe how we arrived at these two values.

#### Efficacy of masks

The efficacy of masks was defined as the size of the reduction in transmission probability during a contact between a susceptible and an exposed individual when a mask is worn by one or both parties. For protecting the healthy wearer, systematic reviews and meta-analyses in other viruses have found mean effect sizes of around 45% in community settings^12^ with a range of approximately 20–80%^12–14^. These should be adjusted downwards to account for different mask types, since cloth masks, the kind most commonly used among the UK public^7^, are less effective than the N95 ventilators used by some study participants^12^. The reviewed studies, mostly case–control, were also subject to potential biases that may have inflated the effect size; for example, people who wear masks may tend to be more careful, such as by washing hands more often and keeping greater distance from cases, than those who do not. However, we must also factor in source control. Experiments have shown face coverings, especially but not exclusively medical masks, to greatly reduce viral shedding and droplet dispersal^8,9^. Taking all of these into consideration, we modelled a mean of 45% with a range of 25–70% as a reasonable estimate of face covering efficacy. This is calculated as the weighted average of one-person masking and both-people masking.

#### Coverage of masks across different contact network layers

##### School contact network layer

Within the model, we had to make assumptions on who may be wearing masks at schools. The education system in the UK consists of 14 school years (age 4–18 years) each of which start in September and finish the following July. There are seven years of primary school, with children entering reception aged 4 and leaving aged 11, followed by 7 years of secondary school. We model school years defined by age bands, noting that some variability may occur in these across different school settings that we don’t account for. The masks policy in place prior to September 2020 was that masks were recommended in corridors and other communal “hot spots’ areas where there is a higher risk of COVID-19 transmission^50^, although this was implemented differently across different schools. Following discussion among the authors and with scientific advisors and policy decision makers in the UK, we decided to model the use of masks by secondary school students only, i.e. the last 7 years of education. This implies that the coverage of masks in the school layer would be at most 50%. We note that this would be reduced if masks were not worn in classrooms and if students’ use of masks were not perfect. The efficacy of face coverings was estimated to be 25–70%, and to account for uncertainty, we modelled two efficacy values of 30% and 60%. Multiplying by a coverage level of 50% we derived an effective coverage of 15% and 30% describing lower and higher effective coverage of masks in schools.

##### Community contact network layer

In our previous work^28^ we assumed that with schools reopening, the society will reopen proportionally to school years going back. This would imply that with schools fully going back, the society would reopen to 90% of its pre-COVID-19 level. However, reports from surveys tracking the increase in contact rates suggest that during July and August the contact rate only marginally increased to ~ 4 contacts per person, which is 36% of the pre-COVID-19 level. This is anticipated to increase with schools reopening but we anticipate not to 90% as modelled before, but likely to around 70% of the pre-COVID-19 level.

In terms of mask usage in the UK, self-reported surveys for August 31 to September 6, 2020 suggest that 64% of Britons “always” and 15% of Britons “frequency” wear mask while in public places^51^. Since we do not model specific parts of the community, e.g. transport network or shops, separately, we use a value slightly larger than the mean coverage level reported in the surveys^51^ and assume that overall adherence to masks in community is around 50%. As before, the efficacy of face coverings was estimated to be 25–70%, and to account for uncertainty, we modelled two efficacy values of 30% and 60% producing effective coverage levels of 15% and 30%.

##### Workplace contact network layer

As of August 2020, face coverings were not mandatory in workplaces across UK. In our previous work^28^, we assumed that with schools reopening, 70% of workplaces would also reopen to all staff. An August 2020 report from the Office for National Statistics suggested that 49% of workers were working from home^45^. We anticipate the proportion of people going back to work would increase after the reopening of schools. We thus assume across scenarios that 60% of the workforce would return with reopening of schools during school term-time, 10% of which would additionally work from home during the school holidays. We also assume that making workplaces COVID-19 secure would reduce transmission in the workplace, and hence model the transmission probability for the workplace layer to be 50% of the pre-COVID-19 level during term time and 40% during school holidays.

### Scenarios

While it is possible in our framework to explore the effect of varying assumptions regarding mask type, effectiveness, compliance, geographical location, setting (school, workplace, community) and other factors that may affect the impact of mask policies, this would require sampling an infeasible number of scenarios, and be of limited value given the standard of evidence available. Instead, under the scenarios of reopening of schools from September 2020, alongside society, and under different levels of TTI strategies, we simulated four scenarios describing two policies on face coverings and two levels of face coverings effective coverage. The four scenarios are modelled by reduction in the transmission probability across layers as described in Table 1.

### Analysis

For each of the four scenarios, we estimated the daily and cumulative numbers of infections until October 23, 2021 for all possible combinations of test and trace levels. Since Covasim is stochastic, we simulated each scenario under 30 different random number seeds, with these random number seeds selected during the calibration process, and in the results we present the median estimates along with ranges corresponding to the upper and lower bounds generated by these 30 seeds.

Across each scenario, we illustrated our results for two test-trace combinations using the current tracing level and two testing levels: August 2020 levels vs scaled-up levels to avoid a second wave. The resurgence of COVID-19 after September 01, 2020 and a second wave is avoided if the median daily number of cases remain below 20,000 infections and the median cumulative number of cases remain below 500,000 over the simulation period.

## Supplementary Information

Supplementary Information.

## Data Availability

The data that support the findings of this study are available from the corresponding author upon reasonable request.

## Code availability

The code used to run all simulations contained in this paper is available from https://github.com/Jasminapg/Covid-19-Analysis.
