# Supplementary material for: Modelling the potential impact of mask use in schools and society on COVID-19 control in the UK
Source: Sci Rep. 2021 Apr 22;11:8747. doi: 10.1038/s41598-021-88075-0 (PMC8062670; doi:10.1038/s41598-021-88075-0)
Supplement: Supplementary file 1 — Supplementary Information. [file 41598_2021_88075_MOESM1_ESM.pdf]

Supplementary material to
Modelling the potential impact of mask use in schools and society on COVID-19 control in the UK

J.Panovska-Griffiths[1,2,3*], C.C. Kerr[4,5], W. Waites[6], R.M. Stuart[7,8], D. Mistry[4], D. Foster[9], D.J. Klein[4], R.M.Viner[10$], C. Bonell[11$],

[1]Department of Applied Health Research, University College London, London, UK
[2]Institute for Global Health, University College London, London, UK
[3]The Queen's College, Oxford University, Oxford, UK
[4]Institute for Disease Modeling, Seattle, WA, USA
[5]School of Physics, University of Sydney, Sydney, NSW, Australia
[6]School of Informatics, University of Edinburgh, UK
[7]Department of Mathematical Sciences, University of Copenhagen, Copenhagen, Denmark
[8]Disease Elimination Program, Burnet Institute, Melbourne, VIC, Australia
[9]Rethink Priorities, Redwood City, CA, USA
[10]UCL Great Ormond St. Institute of Child Health, London, UK
[11]Faculty of Public Health and Policy, London School of Hygiene and Tropical Medicine, London, UK

$ These authors contributed equally
*Corresponding author: j.panovska-griffiths@ucl.ac.uk

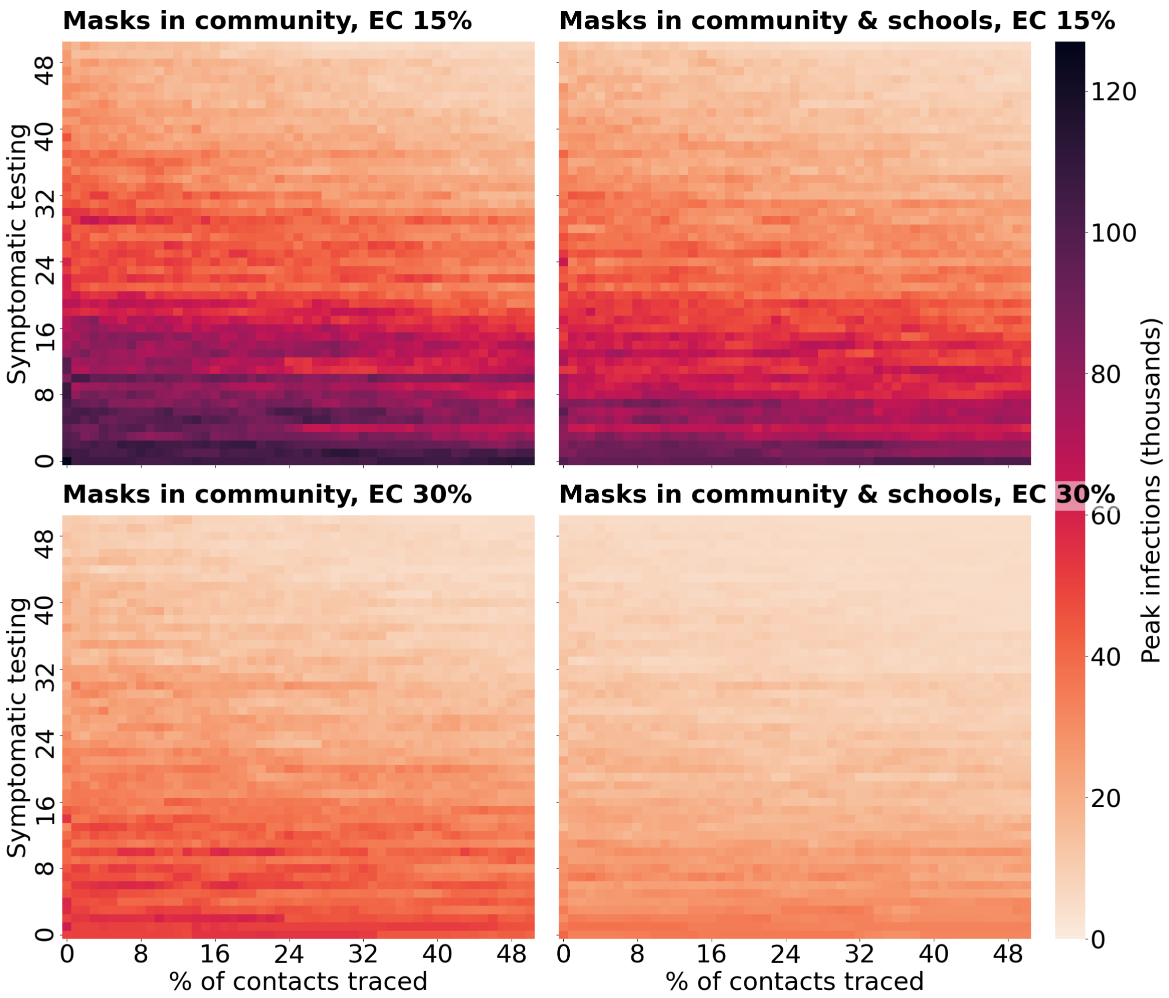

Figure S1: Heatmaps of the peaks of new infections for different trace (x-axis) and test (y-axis) levels across the scenario of mask wearing in parts of community with and without schools masks' wear. Higher values of the peak of new infections are shown in darker shades of red, while lower values are lighter colours. The region of a light orange colour with median daily peak infections below 20,000 represents a region within where the second wave of COVID-19 after September 2020 is avoided with combinations of adequate test-trace and mask usage.

**Calibration of the model**	
Initial seeding of infectious persons on 21/02/2020	1500
β	0.00748
p_s in March	0.009
p_s in April	0.013
p_s in May	0.026
p_s in June	0.0277
p_s in July and August	0.0277
p_{as} in May and June	0.00075

Table S1: Parameters fitted during the calibration.β describes the per-transmission probability, p_s describes the probability of testing for symptomatic people and p_{as} describes the probability of testing for asymptomatic people

Disease Duration Parameters

Parameter	Description	Distribution (mean, std)	Source
s	Length of time after exposure before an individual is infectious (i.e. has begun viral shedding)	$s \sim lognormal\,(4.6, 4.8)$	[1-5]
i_1	Length of time after viral shedding has begun before an individual has symptoms	$i_1 \sim lognormal\,(1, 0.9)$	[1,7]
i_2	Length of time after symptoms appear before they become severe and the person requires critical care	$i_2 \sim lognormal\,(6.6, 4.9)$	[7]
i_3	Length of time after severe symptoms appear before the person requires critical care	$i_3 \sim lognormal\,(3, 7.4)$	[8]
r_a	Recovery time for asymptomatic cases	$r_a \sim lognormal\,(8, 2)$	[6]
r_m	Recovery time for mild cases	$r_m \sim lognormal\,(8, 2)$	[6]
r_s	Recovery time for severe cases	$r_s \sim lognormal\,(14, 2.4)$	[5]
r_c	Recovery time for critical cases	$r_c \sim lognormal\,(14, 2.4)$	[5]

Table S2: Disease duration parameters, in days, used in simulations. This table is modified from [36], which describes the Covasim model in more detail.

Age-linked Disease Parameters

Parameter	0-9	10-19	20-29	30-39	40-49	50-59	60-69	70-79	80+
r_{sus}	0.34	0.67	1.00	1.00	1.00	1.00	1.00	1.24	1.47
p_{sym}	0.50	0.55	0.60	0.65	0.70	0.75	0.80	0.85	0.90
p_{sev}	0.0005	0.0065	0.0072	0.0208	0.03430	0.0765	0.1328	0.2066	0.2457
p_{cri}	0.00003	0.00008	0.00036	0.00104	0.00216	0.00933	0.03639	0.08923	0.1742
p_{death}	0.00002	0.00006	0.00030	0.00080	0.00150	0.00600	0.02200	0.05100	0.09300

Table S3: Age-linked disease parameters used in simulations. This table is borrowed from [9] which describes the Covasim model in more detail. Key: r_{sus}: relative susceptibility to infection; p_{sym}: probability of developing symptoms; p_{sev}: probability of developing severe symptoms (i.e., sufficient to justify hospitalisation); p_{cri}: probability of developing into a critical case (i.e. sufficient to require ICU); p_{death}: probability of death (i.e. infection fatality ratio)

Layer	Mar 23 – May 31	Jun 1 – Jun 14	June 15 – Jul 24	Jul 24 – Aug 31
Households	100%	100%	100%	100%
Schools	2%	21%	36%	2%
Workplaces	20%	40%	50%	30%
Community	20%	40%	50%	50%

Table S4: Scale factors applied to daily "pre-lockdown" SARS-CoV-2 transmission probabilities in households, schools, workplaces prior to September 2020.

References:

1. Lauer, S.A. *et al.* The Incubation Period of Coronavirus Disease 2019 (COVID-19) From Publicly Reported Confirmed Cases: Estimation and Application. *Ann Intern Med*. **172(9),** 577-582 (2020).
2. Pung, R. *et al.* Investigation of three clusters of COVID-19 in Singapore: Implications for surveillance and response measures. *The Lancet.* **395 (10229),** 1039–1046 (2020).
3. Du, Z. *et al.* Serial Interval of COVID-19 among Publicly Reported Confirmed Cases. *Emerg Infect Dis.* **26(6),**1341-1343(2020).
4. Nishiura, H., Linton, N. M. & Akhmetzhanov, A. R. Serial interval of novel coronavirus (COVID-19) infections. *Int J Infect Dis.* **93**, 284–286 (2020).
5. Verity, R. *et al.* Estimates of the severity of coronavirus disease 2019: A model-based analysis. *The Lancet Infect Dis*. 20(6), 669-677 (2020).
6. Wölfel, R. *et al*. Virological assessment of hospitalized patients with COVID-2019. *Nature*. **581(7809**), 465-469 (2020).
7. Linton, N.M. *et al.* Incubation period and other epidemiological characteristics of 2019 novel coronavirus infections with right truncation: A statistical analysis of publicly available case data. *J Clin Med*. **9(2),** 538 (2020).
8. Wang, D. *et al.* Clinical characteristics of 138 hospitalized patients with 2019 novel coronavirus-infected pneumonia in Wuhan, China. *JAMA*. **323(11),** 1061–1069 (2020).
9. Kerr, C.C. et al. Covasim: an agent-based model of COVID-19 dynamics and interventions. Preprint at https://doi.org/10.1101/2020.05.10.20097469 (2020).
